# The *Drosophila* Mitochondrial Translation Elongation Factor G1 Contains a Nuclear Localization Signal and Inhibits Growth and DPP Signaling

**DOI:** 10.1371/journal.pone.0016799

**Published:** 2011-02-25

**Authors:** Catherine Trivigno, Theodor E. Haerry

**Affiliations:** 1 Center for Molecular Biology and Biotechnology, Florida Atlantic University, Boca Raton, Florida, United States of America; 2 Department of Biological Sciences, Florida Atlantic University, Boca Raton, Florida, United States of America; Skirball Institute of Biomolecular Medicine - New York University Medical Center, United States of America

## Abstract

Mutations in the human mitochondrial elongation factor G1 (EF-G1) are recessive lethal and cause death shortly after birth. We have isolated mutations in *iconoclast* (*ico*), which encodes the highly conserved *Drosophila* orthologue of EF-G1. We find that EF-G1 is essential during fly development, but its function is not required in every tissue. In contrast to null mutations, missense mutations exhibit stronger, possibly neomorphic phenotypes that lead to premature death during embryogenesis. Our experiments show that EF-G1 contains a secondary C-terminal nuclear localization signal. Expression of missense mutant forms of EF-G1 can accumulate in the nucleus and cause growth and patterning defects and animal lethality. We find that transgenes that encode mutant human EF-G1 proteins can rescue *ico* mutants, indicating that the underlying problem of the human disease is not just the loss of enzymatic activity. Our results are consistent with a model where EF-G1 acts as a retrograde signal from mitochondria to the nucleus to slow down cell proliferation if mitochondrial energy output is low.

## Introduction

Birth defects due to problems in mitochondrial function affect a large number of children. Many are associated with mutations in genes involved in the translation of proteins in mitochondria required for oxidative phosphorylation, which supplies approximately 90% of the energy used by eukaryotic cells. There are five protein complexes in mitochondria that are required for this process: four comprise the mitochondrial respiratory chain and complex V functions as ATP synthase [1]. While all five complexes contain proteins derived from nuclear genes, complexes I, III, IV, and V also contain a total of thirteen proteins encoded within the mitochondrial DNA [2]. The biosynthesis of these proteins requires several nuclear-encoded factors, which are translated in the cytoplasm and imported into mitochondria [3]. Mutations in these factors severely impact mitochondrial function, since they impede the synthesis of all thirteen proteins encoded in the mitochondrial DNA and affect all but complex II. For example, the fatal pediatric disorder Combined Oxidative Phosphorylation Deficiency 1 (COXPD1) is caused by mutations in a single gene that encodes the human mitochondrial translation elongation factor G1 (EF-G1). Children with mutations in EF-G1 suffer from early onset Leigh's syndrome, lactic acidosis, exhibit severe neurological defects, and typically die shortly after birth due to liver failure [4], [5], [6]. Currently, the underlying problem of this disease is not well understood. Previous findings have shown that cells in tissues affected by the disease exhibit reduced levels of EF-G1 protein in mitochondria [5]. However, it is not clear whether the mutant forms of EF-G1 are degraded or not imported into mitochondria.

In this report, we describe the identification and characterization of mutations in the *Drosophila* orthologue of EF-G1, *iconoclast* (*ico*). The *ico* locus was identified in suppressor and enhancer screens for mutations that interact with TGF-beta signaling. Our analysis reveals that *ico* is an essential gene but not required in every tissue. Interestingly, missense alleles exhibit a much more severe phenotype than null mutations. When expressed from transgenes, we find that mutant EF-G1 proteins, which encode a secondary C-terminal nuclear signal sequence, are not degraded and can translocate to the nucleus and inhibit growth and disrupt patterning. Taken together, out results are consistent with a model where EF-G1 may function as a retrograde signal from mitochondria to the nucleus to prevent cell proliferation, if mitochondrial ATP synthesis is too low.

## Results

### EMS-mutations in *ico* interact with DPP signaling

**Figure 1:**
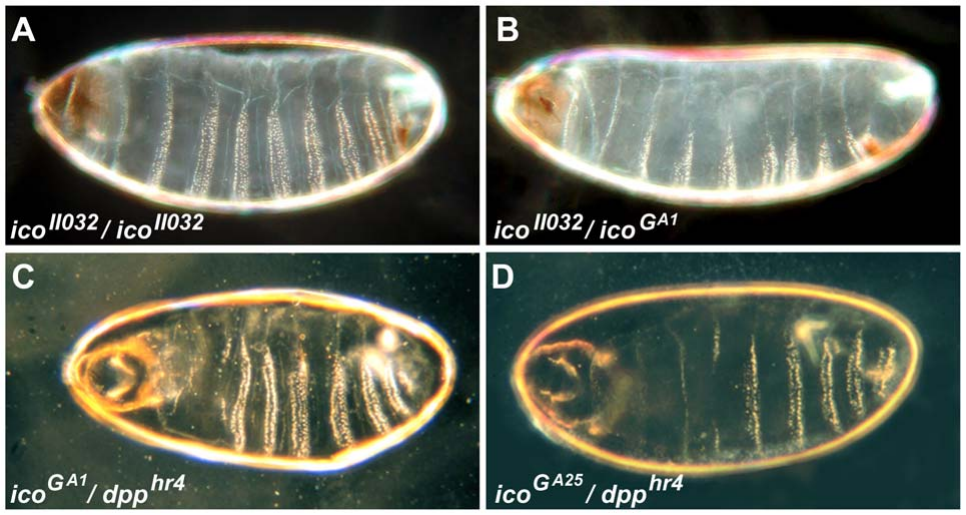
EMS-mutant *ico* alleles are embryonic lethal and interact with DPP signaling. (A–D) Cuticle preparations of dead embryos. (A and B) Homozygous or transheterozygous combinations of the *ico* alleles *II032*, *G^A1^*, *G^A25^*, *and B^A18^* die as embryos with head involution defects. (C and D) Animals heterozygous for *ico* EMS-mutant alleles are embryonic lethal in combination with *dpp^hr4^*, indicating that they exhibit reduced DPP signaling.

Polypeptide cytokines of the TGF-beta family contribute to a wide range of developmental and physiological functions in higher eukaryotes. Among many functions, this diverse group of signaling molecules promotes growth and controls cell death [7]. In a genetic screen for mutations that interact with an activated form of the TGF-beta type I receptor Thick Veins (TKV), we isolated several genes that suppressed the growth and pattern defects of excess TKV signaling in wings [8], [9]. When tested in a second maternal enhancer screen, only a few these mutations were able to reduce signaling of the ligand Decapentaplegic (DPP) and cause embryonic lethality in combination with the allele *dpp^hr4^* [10]. Among the mutations that strongly interacted with DPP signaling in both screens we identified alleles of the downstream transcription factors *mad* and *medea*, the Glypican *dally*, and the not characterized mutation *II032*. This mutation was originally isolated in a large scale mutagenesis screen for recessive embryonic lethal genes [11]. Since the gene affected by this mutation had not been identified, we named this locus, which is located on the left arm of chromosome 2 (L2), *iconoclast* (*ico*). We tested, whether mutations from a different mutagenesis screen that were mapped to this location failed to complement *II032* mutants, and identified three additional alleles of *ico*, *G^A1^*, *G^A25^*, *and B^A18^* [12]. Homozygous and transheterozygous combinations of all four *ico* alleles are embryonic lethal and die within 24 hours with head involution defects (Figure 1A and B). In addition, heterozygous mutants of all four alleles cause lethality in combination with *dpp^hr4^*, indicating that they reduce DPP signaling (Figure 1C and D).

### *Ico* encodes *CG4567*, the *Drosophila* orthologue of the mitochondrial elongation factor G1

**Figure 2:**
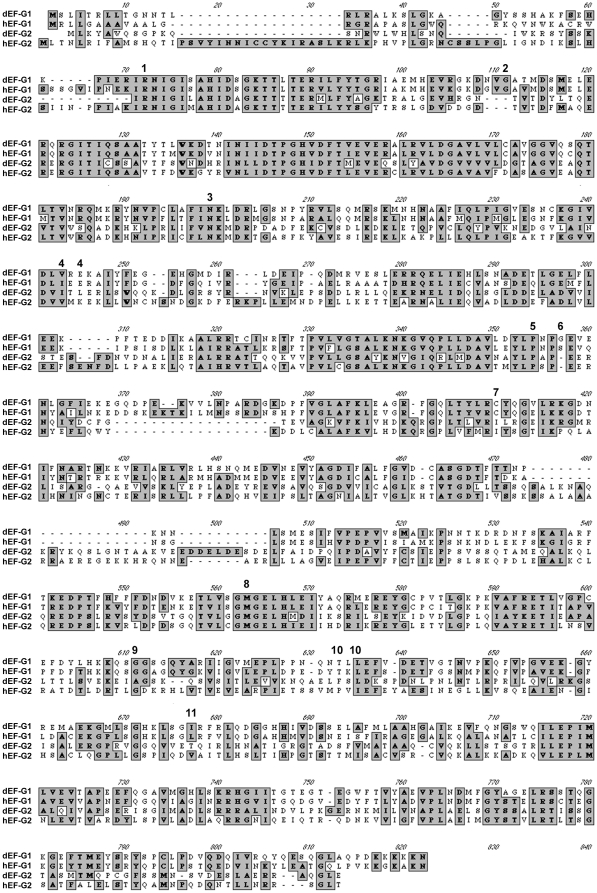
Sequence alignment of *Drosophila* and human EF-G1 and EF-G2 proteins. Sequence alignment of the *Drosophila* and human EF-G1 and EF-G2 proteins (dEF-G1, hEF-G1, dEF-G2, hEF-G2). The positions of the identified fly and human *EF-G1* mutations shown in Figure 3 are indicated by numbers (1–11) [4], [5], [6].

**Figure 3:**
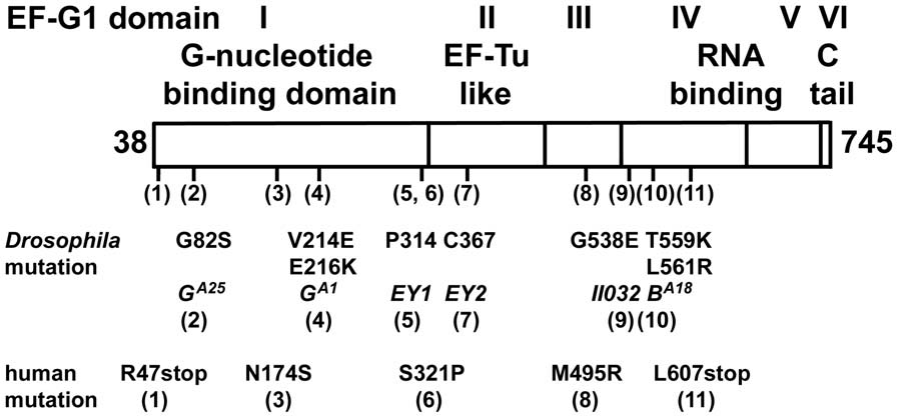
The identified mutations in EF-G1 are located in various domains. Similarities between EF-G1 and bacterial G-factors indicate that the proteins likely share a similar three-dimensional structure [20]. The first and largest domain of EF-G1 is its GTP-GDP binding domain. Six of the identified mutations are located in this domain. The second domain shares homology to the elongation factor EF-Tu. Domains IV and V show similarities with other RNA binding proteins, and structural analysis suggests that Domains III, IV, and V form an interface mimicking the shape of a tRNA that is used to interact with ribosomes [13]. In addition to bacterial G-factors and mitochondrial EF-G2 proteins, EF-G1 contains a positively charged C-terminal tail. The deletion EY1 removes 1888 nucleotides, which creates a stop codon six amino acids after P314 and encodes a truncated protein that only consists of the first domain. Similarly, deletion EY2 removes 3663 nucleotides, which creates a stop codon 19 residues past C367 and encodes a protein truncated within domain II.

Complementation tests with well-defined deficiencies on L2 indicated that the mutations might be located within *CG4567*, which encodes the *Drosophila* orthologue of the mitochondrial elongation factor G1 (dEF-G1; Figure 2). To identify the gene, we generated imprecise excisions of various P-elements including *EY05983*, which is located 3′ of *CG4567*. We isolated two deletions, *ico* ***^Del EY1^*** and *ico* ***^Del EY2^***, which failed to complement *ico* alleles. Subsequent sequencing revealed that the EMS-induced mutations contained single or double missense mutations in various regions of *CG4567* (Figures 2 and 3). Both deletions remove the C-terminal RNA binding domains of *CG4567* (Figure 3), which according to structural comparison mimic the shape of a tRNA and are used to interact with ribosomes [13]. Thus, the deletion mutations most likely represent null mutations, since the resulting truncated proteins are not expected to form functional complexes with ribosomes. Although the allele *ico* ***^Del EY2^*** also lacks a portion of the neighboring gene *CG13784*, the phenotypes of the two deletions are identical. Interestingly, homozygous or transheterozygous animals of the two deletions are not embryonic lethal like the four missense mutations but develop for two additional days to the early third instar larval stage, where they arrest development and die several days later. Animals with a combination of a missense mutation and a deletion die shortly after embryogenesis as malformed first instar larvae. In contrast to missense mutations, the two deletions also do not interact with DPP signaling in both genetic screens. Taken together, our results show that *ico* alleles are mutations in *CG4567* and that missense mutations that encode full-length EF-G1 proteins cause a more severe phenotype than null mutations.

### Rescue of *ico* mutants with human EF-G1 transgenes

To further confirm that *ico* alleles are mutations in *CG4567*, we generated transgenic flies with the *CG4567* coding region under the control of the upstream activating sequences of GAL4 to perform mutant rescue experiments (*pUAST* and *pUAST2* [14], [15]). In addition, we made transgenic flies that express the highly conserved human EF-G1 orthologue (hEF-G1, Figure 2), the CG4567-G538E protein made in *II032* mutants (Figure 3), the previously characterized human mutant proteins hEF-G1-N174S and hEF-G1-S321P (Figure 3), as well as the *Drosophila* orthologue of EF-G2, CG31159 (dEF-G2, Figure 2). When expressed ubiquitously with a combination of the *armadillo* and *daughterless* GAL4 drivers (*arm+da*-GAL4), we find that the fly and human wild type transgenes are able to rescue close to 100% of *ico* mutant animals (Table 1, row 1, 2, and 6). This result confirms that *ico* alleles are mutations in *CG4567* and that the functions of the EF-G1 proteins are conserved between flies and humans. Compared to the wild type transgenes, the CG4567-G538E mutant form retains little activity, since ubiquitous expression of this protein is unable to rescue a single mutant animal to adulthood (Table 1, row 4). In contrast, more than 80% of expected mutant animals are rescued by the human mutant form N174S [4], indicating that this mutant form retains a high level of functional activity in flies (Table 1, row 7). However, we noticed that the first animals rescued by this transgene hatch 3 to 5 days later than heterozygous animals or animals rescued with wild type transgenes. A delay of at least 5 days is also seen in mutant animals that are rescued by transgenes that encode the mutant hEF-G1-S321P [5]. Compared to N174S, only 40% of the expected animals survive to adulthood (Table 1, row 8), suggesting that the S321P mutant proteins retain less activity than N174S. In contrast to the fly and human *EF-G1* transgenes, no animals were rescued with a single or two recombined transgenes that express the *Drosophila* EF-G2 protein (Table 1, row 9 and 10). As in tissues of COXPD1 patients [5], it appears that overexpression of EF-G2 cannot compensate for the absence of EF-G1.

**Table 1:** Rescue of *ico* mutants by fly and human EF-G1 proteins.

	Adults	Rescue
*UAS-CG4567/w*; *ico* ***^GA1^*** * ico* ***^Del EY2^***; *+/arm+da-GAL4* *UAS-CG4567/w*; *CyO/ico* ***^Del EY2^***; *+/arm+da-GAL4*	6770	67/70 = 96%
*UAS-CG4567/w*; *ico* ***^Del EY1^*** */ico* ***^Del EY2^***; *+/arm+da-GAL4* *UAS-CG4567/w*; *CyO/ico* ***^Del EY2^***; *+/arm+da-GAL4*	7270	72/70 = 103%
*UAS-TP-GFP-CG4567/w*; *ico* ***^Del EY1^*** */ico* ***^Del EY2^***; *+/arm+da-GAL4* *UAS-TP-GFP-CG4567/w*; *CyO/ico* ***^Del EY2^***; *+/arm+da-GAL4*	6570	65/70 = 93%
*UAS-CG4567-G538E/w*; *ico* ***^Del EY1^*** */ico* ***^Del EY2^***; *+/arm+da-GAL4* *UAS-CG4567-G538E/w*; *CyO/ico* ***^Del EY2^***; *+/arm+da-GAL4*	0102	0/102 = 0%
*UAS-CG4567-ΔCstop/w*; *ico* ***^Del EY1^*** */ico* ***^Del EY2^***; *+/arm+da-GAL4* *UAS-CG4567-ΔCstop/w*; *CyO/ico* ***^Del EY2^***; *+/arm+da-GAL4*	6166	61/66 = 92%
*UAS-hEF-G1/w*; *ico* ***^Del EY1^*** */ico* ***^Del EY2^***; *+/arm+da-GAL4* *UAS-hEF-G1/w*; *CyO/ico* ***^Del EY2^***; *+/arm+da-GAL4*	6773	67/73 = 92%
*UAS-hEF-G1-N174S/w*; *ico* ***^Del EY1^*** */ico* ***^Del EY2^***; *+/arm+da-GAL4* *UAS-hEF-G1-N174S/w*; *CyO/ico* ***^Del EY2^***; *+/arm+da-GAL4*	7492	74/92 = 80%
*w/w*; *ico* ***^Del EY1^*** */ico* ***^Del EY2^***; *UAS-hEF-G1-S321P/arm+da-GAL4* *w/w*; *CyO-TM6B/ico* ***^Del EY2^***; *arm+da-GAL4*	3895	38/95 = 40%
*UAS-CG31159/w*; *ico* ***^Del EY1^*** */ico* ***^Del EY2^***; *+/arm+da-GAL4* *UAS-CG31159/w*; *CyO/ico* ***^Del EY2^***; *+/arm+da-GAL4*	0111	0/111 = 0%
*w/w*; *ico* ***^Del EY1^*** */ico* ***^Del EY2^***; *UAS-CG31159 (2x)/arm+da-GAL4* *w/w*; *ico* ***^Del EY2^***; *CyO-TM6B/arm+da-GAL4*	097	0/97 = 0%

Females with *ico* mutations and fly or human transgenes on the X or third chromosome *(UAS-EF-G1/UAS-EF-G1*; *ico/CyO*; *+/+* or *w/w*; *ico*; *UAS-EF-G1/CyO-TM6B)* were crossed to males with *ico* mutations and the ubiquitous GAL4 drivers *armadillo*-GAL4 and *daughterless*-GAL4 balanced over chromosomes with dominant curly wing and tubby body markers *(w/Y*; *ico* ***^Del EY2^***; *arm+da-GAL4/CyO-TM6B)*. The numbers of the rescued homozygous *ico* mutant females, which have straight wings and are not tubby, were compared to their heterozygous *ico* sisters, which have curly wings. (Row 3) *TP-GFP-CG4567* transgenes that express a GFP-tagged *Drosophila* EF-G1 protein inserted after the N-terminal target sequence (TP) encode a functional protein that can rescue *ico* mutants. (Row 4, 9, and 10) Transgenes that express the *II032* mutant EF-G1 or *Drosophila* EF-G2 (CG31159) proteins do not rescue *ico* mutants. (Row 5) *CG4567-ΔCstop* is a transgene with a premature stop codon that lacks the C-terminal tail. (Row 7 and 8) The adults rescued with *hEF-G1-N174N* and *hEF-G1-S321P* hatched at least 3-5 days later than their siblings did. (Row 8 and10) The number of the animals rescued with the *hEF-G1-S321P* or *CG31159* transgenes was compared to curly and tubby animals with the darker eye color of the *arm+da-GAL4*.

### The function of EF-G1 is not required in the eye

The deletions *ico* ***^Del EY1^*** and *ico* ***^Del EY2^*** remove a large portion of the EF-G1 C-terminal region that is likely essential for its function [16]. However, these null mutations live substantially longer than flies with alleles encoding full-length proteins with missense mutations, suggesting that the proteins derived from missense mutations cause additional defects. To further investigate differences between deletion and missense mutations, we compared the fates of homozygous clones of *ico* mutants in somatic cells of the eye, a tissue that is not essential for viability. Using the FRT/Flipase system [17], we recombined *ico* mutants with an FRT containing transgene close to the centromere of 2 L. When the site-specific recombination enzyme Flipase is expressed under the control of *eyeless* regulatory sequences in eyes of a heterozygous animal with two FRT sites at the same position on both sister chromosomes, somatic recombination of the chromosomal arms can occur during mitosis, resulting in daughter cells that contain two wild type or two mutant copies. To distinguish the homozygous *ico* clones generated by this process from the homozygous wild type clones and non-recombined heterozygous cells, this experiment is performed in a *white* background with a mini-*white* transgene on 2 L to mark the heterozygous *ico* cells with red pigment. Since the *ico* mutant clones could exhibit reduced proliferation rates and might be out-competed by the wild type and heterozygous cells, a recessive cell-lethal mutation is located on 2 L of the sister chromosome to eliminate homozygous clones that are wild type for *ico*. Since the Flipase is located on the X-chromosome, only females will undergo recombination in the eye. Heterozygous cells that do not recombine express the mini-*white* transgene and are red. Homozygous twin clones of the sister chromosome should die due to the cell-lethal mutation. Homozygous clones of *ico* mutants are white, since they do not inherit the mini-*white* transgene.

**Figure 4:**
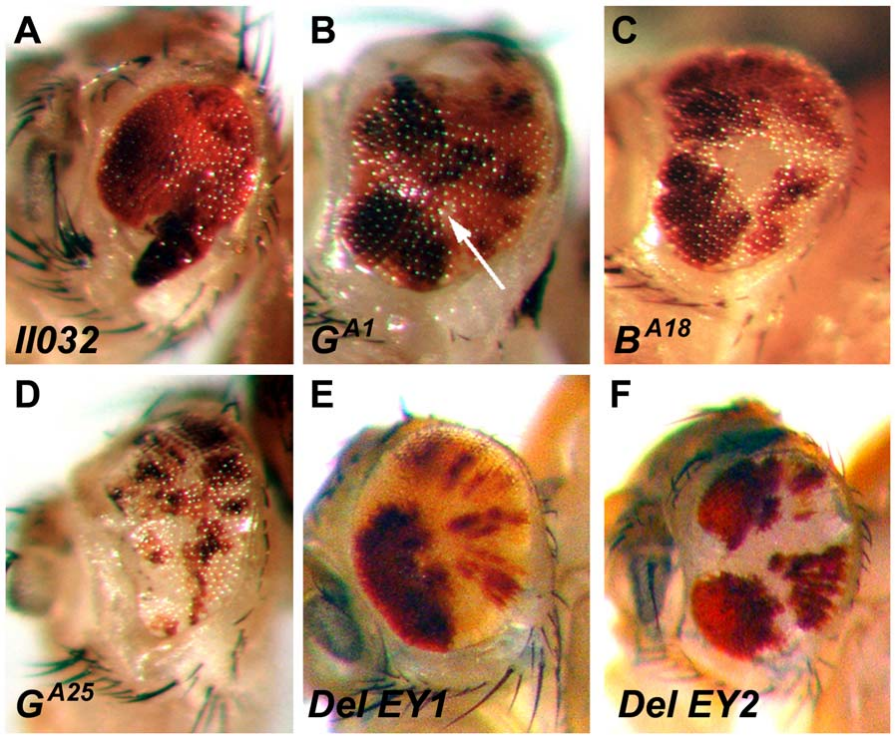
Clonal analysis of *ico* mutants in the eye. Clones of homozygous *ico* alleles were generated with *eyeless*-Flipase in the eyes of females. Homozygous *ico* clones do not contain eye pigment and are white. Pigmented cells are heterozygous for *ico*. (A) Clones of the mutation *II032* do not survive to adulthood. (B–D) Clones of the other three missense mutations exhibit a small numbers of white cells. In contrast, homozygous clones of the two null mutations develop normally (E and F), indicating that EF-G1 function is not required in the eye and that clones expressing C-terminally truncated proteins do not affect growth and patterning. The cause for the black patches of cells that can be clearly seen in *G^A1^*, *B^A18^*, *and G^A25^* but are also present in the other alleles is not entirely clear. However, their presence does not affect the interpretation of the results. See text for details.

The absence of white cells in heterozygous *II032* animals suggests that homozygous clones of this missense mutation do not survive to adulthood (Figure 4A). The size of the eyes is reduced and patterning is disrupted, since the recombined cells die and only non-recombined cells survive and proliferate. Compared to *II032*, a few small white clones appear in the eyes of *G^A1^*, *B^A18^*, *and G^A25^* mutant animals (Figure 4B-D, arrow), indicating that these mutations severely affect cell proliferation or survival rates. *G^A25^* mutant animals exhibit the largest number of white cells, suggesting that it is the weakest of the missense mutations (Figure 4D). In contrast to missense mutations, the eyes of the deletion mutations *ico* ***^Del EY1^*** and *ico* ***^Del EY2^*** show no pattern defects and contain large areas of white homozygous clones (Figure 4E and F). The finding that cellular clones of *ico* mutants that almost certainly represent null mutations develop normally and do not disrupt patterning of the eyes indicates that the function of *Drosophila* EF-G1 is not required in the eye and possibly other tissues. Since elongation is an essential process and all eukaryotes encode a second highly conserved mitochondrial G factor, EF-G2, it is possible that *Drosophila* EF-G2 can compensate for the lack of EF-G1 in certain tissues like the eye. Taken together, our results demonstrate that the presence of *ico* missense mutations has deleterious effects on cell proliferation or survival even in tissues where *Drosophila* EF-G1 is apparently not required.

It is unclear why clusters of black cells appear particularly in the eyes of *G^A1^*, *B^A18^*, *and G^A25^* mutant animals (Figure 4B–D). The latter three mutations were all induced in a *black* and *cinnabar* background [12]. However, since darker spots are also present in the eyes of the other alleles, they may represent twin clones. This is only possible, if the cell-lethal gene on the sister chromosome is not entirely lethal. Since two copies of the mini-*white* gene cannot cause this color, it can only be explained if an additional undisclosed recessive eye color marker is present on that chromosome. Whatever the cause is for these patches, they do not affect the interpretation of the presented results.

### Expression of an EF-G1 missense mutant form causes growth and patterning defects in wings

**Figure 5:**
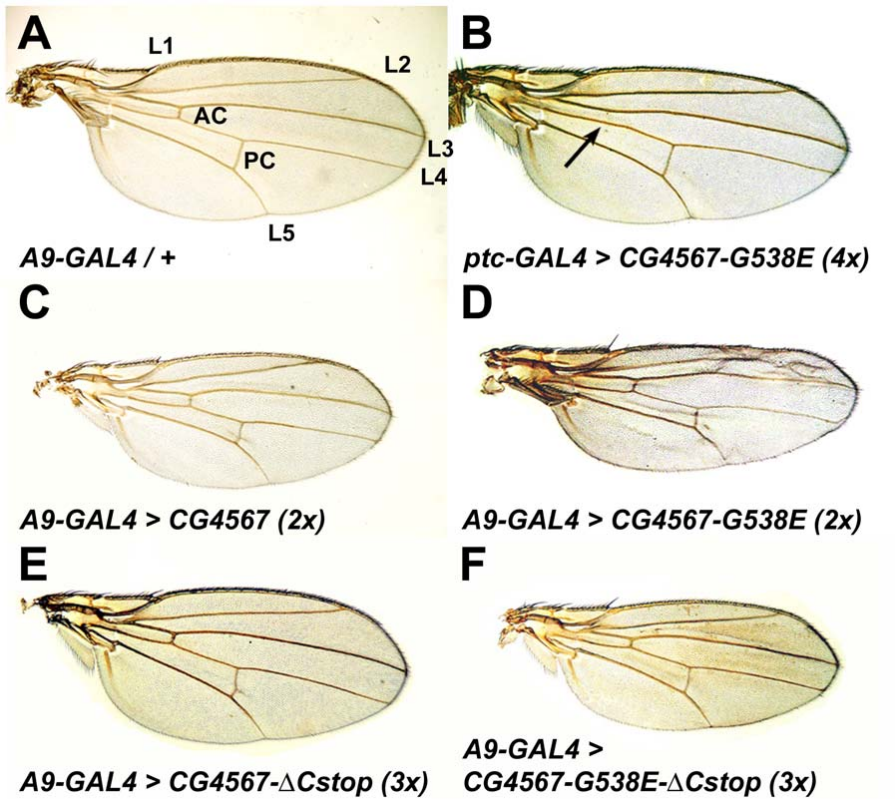
Expression of mutant EF-G1 forms reduces growth and affects patterning in wings. (A) Heterozygous *A9*-GAL4/+ female wing with wild type patterning. Longitudinal veins L1-L5 and the anterior and posterior crossveins are indicated (AC, PC). (B) Wings that express four *CG4567-G538E* transgenes encoding the EF-G1 protein of *II032* mutants with *ptc*-GAL4 show normal proportions but exhibit reduced growth between L3 and L4 where the mutant protein is expressed. They also lack the anterior crossvein (arrow). (C) Overexpression of two wild type *ico* transgenes in wings using *A9*-GAL4 does not affect patterning but results in smaller wings. (D) In comparison, ubiquitous expression of two *CG4567-G538E* transgenes reduces growth and interferes with proper vein formation. (E) Expression of three EF-G1 transgenes that lack the C-terminal tail has no apparent effects on growth or patterning. (F) Compared to two full-length mutant transgenes (D), expression of three tailless mutant CG4567-G538E transgenes results in no major pattern defects but reduces the size of wings.

To further investigate the deleterious effects of EF-G1 missense mutant proteins, we expressed the *II032* mutant form *CG4567-G538E* in various tissues. While animals that ubiquitously express two wild type *ico* transgenes with *daughterless*-GAL4 develop normally, we find that wild type animals that express two copies of *CG4567-G538E* all die before pupation, further supporting the idea that full-length mutant EF-G1 proteins can cause additional defects. When expressed in wings, a tissue that is not essential for viability, we find that *CG4567-G538E* transgenes cause growth and pattern defects. Compared to wild type wings (Figure 5A), wings that express two or four *CG4567-G538E* transgenes under the control of *ptc*-GAL4 exhibit normal proportions but show reduced growth in the area between longitudinal veins 3 and 4 where the mutant proteins are expressed (Figure 5B). In addition, the mutant transgenes also prevent the formation of the anterior crossvein in this area (Figure 5B, arrow). When expressed ubiquitously with the wing driver *A9*-GAL4, two copies of *CG4567-G538E* clearly disrupt wing growth, which leads to patterning defects such as ectopic vein formation and the merging of longitudinal veins (Figure 5D). In comparison, we noticed that ubiquitous expression of two wild type *CG4567* transgenes also reduces growth (Figure 5C). Although we did not quantify the average reduction in growth, the major difference between wild type and mutant transgenes is the absence of pattern defects in wings that express wild type transgenes.

One major structural difference between EF-G1 and EF-G2 is the presence of a short positively charged C-terminal tail that is absent in EF-G2 (Figure 2). To assess its role in growth inhibition, we generated and expressed EF-G1 proteins that lack the C-terminal tail. We find that expression of three *UAS-CG4567-ΔCstop* transgenes have no significant effects on wing growth and patterning (Figure 5E). This result is not due to the lack of enzymatic function, since transgenes that encode the tailless EF-G1 protein are still able to rescue *ico* mutants (Table 1, row 5). Similarly, compared to two full-length mutant transgenes (Figure 5D), wings that express three mutant transgenes without the C-tail show less severe growth defects and no major vein patterning defects (Figure 5F). Unlike two copies of the full-length mutant transgenes, we also find that expression of three copies of the tailless mutant transgenes in all tissues with *daughterless*-GAL4 does not cause developmental arrest and lethality. Finally, we would like to note that overexpression of four copies of EF-G2 does not result in lethality or any wing phenotype (data not shown). Taken together, these results indicate that the unique C-terminal tail of EF-G1 contributes to the growth and pattern defects caused by EF-G1 missense mutant proteins.

### The C-terminal tail of EF-G1 functions as a nuclear localization signal

Previous experiments in tissues of human patients have shown that the underlying problem of the COXPD1 disease is caused by the absence of mutant EF-G1 protein in affected tissues. In the case of the human S321P mutation, the mutant protein cannot be detected in mitochondria of the liver and muscles [5]. In contrast, reduced but significant amounts are still present in mitochondria of the heart, a tissue that is not affected by the disease. The authors favored a model where reduced protein levels are the result of decreased stability and consequent degradation of the mutant EF-G1 proteins. However, human mutant proteins show significant activity in rescue experiments, indicating that they are reasonably stable. If EF-G1 proteins encoded by missense mutations are stable, absent from mitochondria in certain tissues, and can cause growth and pattern defects, it is possible that the observed defects are the result of EF-G1 accumulation outside of mitochondria.

To investigate the subcellular localization of wild type and mutant EF-G1 proteins, we generated constructs with epitope tags. We modified a *pUAST* vector to allow expression of GFP and GST-tagged EF-G1 and EF-G2 proteins with or without the mitochondrial target protein sequence (TP) in flies and Schneider 2 (S2) tissue culture cells (NheI, TP, MssI, GFP/GST, NaeI, protein of interest, XbaI, BglII). The finding that a transgene that encodes a GFP- tagged *Drosophila* EF-G1 protein can rescue *ico* mutants shows that insertion of a tag at this location does not disrupt mitochondrial targeting and the activity of the protein (Table 1, row 3). If the TP sequence is omitted, the coding region initiates from a start codon within GFP or GST. Consequently, such fusion proteins should not be imported into mitochondria. If no protein sequence is inserted at the NaeI site, a stop codon terminates the coding sequence of GFP or GST.

**Figure 6:**
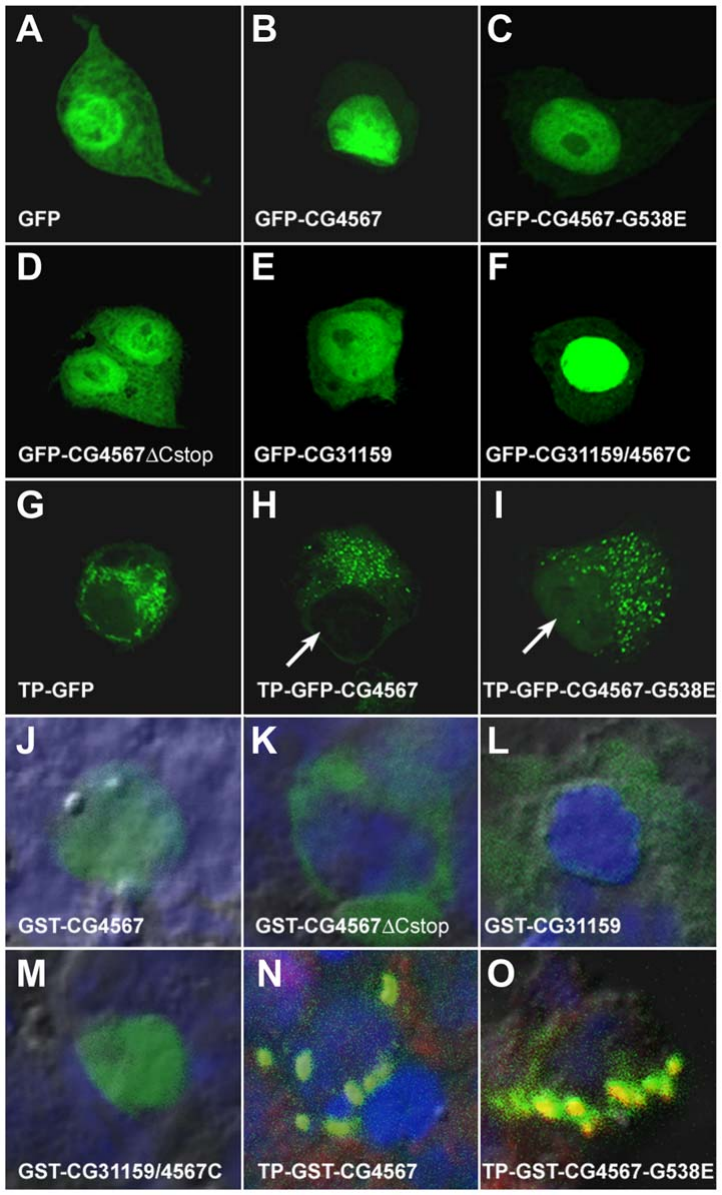
The C-terminal tail of EF-G1 functions as a nuclear localization signal. (A–I) Subcellular localization of GFP tagged EF-G1 (CG4567) and EF-G2 (CG31159) proteins in living S2 cells. (A) GFP is found in the cytoplasm and the nucleus. (B) GFP-tagged EF-G1 that lacks the N-terminal mitochondrial target sequence predominantly localizes to the nucleus. (C) The mutant CG4567-G538E also mainly localizes to the nucleus. (D) In contrast, when the C-terminal tail of EF-G1 is removed, the GFP-tagged protein is found again in the cytoplasm. (E) Similarly, *Drosophila* EF-G2 is cytosolic and nuclear. (F) Addition of the CG4567 C-tail to EF-G2 enhances its nuclear localization. (G) Addition of the N-terminal mitochondrial target protein sequence (TP) of EF-G1 results in punctate GFP staining. (H) A similar punctate pattern is seen with a GFP-tagged EF-G1 protein that contains the TP. (I) While punctate staining is also seen with the mutant CG4567-G538E, one can detect a faint signal of the protein in the nucleus (arrow, compare to H). (J–O) Co-localization of GST-tagged EF-G1 and EF-G2 proteins (green) with DAPI (blue, nucleus) and MitoTracker (red, mitochondria) in fixed S2 cells. (J) A GST-tagged EF-G1 protein without the TP exclusively localizes to the nucleus. (K) Removal of the C-tail of EF-G1 changes the subcellular localization from the nucleus to the cytoplasm. (L) Unlike EF-G1, EF-G2 without the TP is found in the cytoplasm. (M) Addition of the EF-G1 C-tail to EF-G2 alters the subcellular localization from the cytoplasm to the nucleus. (N) A GST-tagged EF-G1 protein with the N-terminal TP co-localizes with MitoTracker in mitochondria that aggregate due to fixation (green+red = yellow). (O) The mutant TP-CG4567-G538E also localizes to mitochondrial aggregates (yellow). However, in contrast to the wild type protein, significant amounts of the mutant protein are also detected outside of mitochondria (green).

To express the fusion proteins in S2 cells, the *pUAST2* constructs were co-transfected with a plasmid that expresses GAL4 under the control of the *actin5C* promoter and enhancer. Four days after transfection, we were able to detect robust expression of GFP without a TP in the cytosol as well as in the nucleus of S2 cells using a confocal microscope (Figure 6A). Compared to GFP alone, expression of GFP-CG4567 or the mutant GFP-CG4567-G538E without the TP is predominantly nuclear (Figure 6B and C). In contrast, removal of the C-tail reverts to a mixed cytosolic and nuclear expression pattern (Figure 6D). An EF-G2 fusion protein (GFP-CG31159) that lacks the TP shows a similarly mixed expression pattern (Figure 6E). However, when the tail of CG4567 is added to the C-terminus of CG31159, we again observe a predominantly nuclear localization of the protein (Figure 6F). When the TP is added to GFP or GFP-CG4567, the expression pattern dramatically changes to a punctate pattern that likely corresponds to mitochondria (Figure 6G and H), since TP-GFP-CG4567 encoding transgenes are able to rescue *ico* mutant flies (Table 1). A similar pattern is seen with the mutant TP-GFP-CG4567-G538E (Figure 6I). However, in contrast to the wild type TP-GFP-CG4567 protein, a faint signal of the mutant protein can be detected in the nucleus (Figure 6H and I, arrows). The finding that only the mutant protein can be detected in the nucleus suggests that the presence of the mutant proteins affects the efficiency of EF-G1 import into mitochondria in wild type cells.

Since GFP by itself can localize to the nucleus, we used proteins tagged with the bacterial GST to further supplement our subcellular localization analysis. Unlike GFP, GST-tagged proteins are monitored with an anti-GST-FITC antibody (green). For this procedure, the cells have to be fixed, which leads to cells adhering to each other, making it more difficult to monitor single cells. Using DAPI (blue) and MitoTracker (red) as marker for the nucleus and mitochondria respectively, we find that GST-CG4567 without the TP exclusively localizes to the nucleus (Figure 6J). Nuclear localization is completely lost, if the C-tail of EF-G1 is removed (Figure 6K). On the other hand, GST-CG31159 without the TP is entirely cytoplasmic but shifts to the nucleus if the CG4567 C-tail is added (Figure 6L and M). In the presence of the TP, the GST-CG4567 co-localizes predominantly with mitochondria that form aggregates due to fixation (Figure 6N, green+red = yellow). While TP-GST-CG4567-G538E is also imported into mitochondria (Figure 6O, yellow), a significantly larger amount of the mutant protein is found outside of mitochondria if compared to the wild type protein (compare Figure 6N and O, green). Taken together, our analysis of GFP and GST fusion proteins shows that EF-G1 has two localization signals, an N-terminal mitochondrial target signal and a C-terminal nuclear signal. In the presence of both signals, wild type EF-G1 proteins are imported into mitochondria and no significant amounts are detected in the nucleus (Figure 6H and N). The proteins expressed by the missense mutant transgenes used in this study are not degraded and similarly stable as the wild type forms. However, compared to the wild type protein (Figure 6H and N), the mutant form is not only imported into mitochondria but also accumulates outside of mitochondria, where it can translocate into the nucleus (Figure 6I and O).

## Discussion

### The defects caused by *EF-G1* missense mutations correlate with the presence of a nuclear localization signal

The characterization of mutations in the *Drosophila* mitochondrial translation elongation factor G1 reveals that, as in humans, it is an essential gene. Animals homozygous for *ico* null mutations are able to survive on maternal contribution to the larval stages but fail to initiate the rapid growth phase during the third larval stage, which is not surprising given the role of EF-G1 in mitochondrial biosynthesis and the high energy demands during that phase. In contrast, animals with EF-G1 missense mutations die prematurely during embryogenesis, indicating that alleles that express full-length mutant EF-G1 proteins exhibit gain-of-function phenotypes. The finding that clones of missense mutants in the eye rarely survive while clones of deletion mutants develop normally further supports this notion. In addition, mutant *ico* transgenes cause larval lethality when overexpressed in all tissues and inhibit growth and disrupts patterning when expressed in wings. Taken together, these results suggest that full-length mutant EF-G1 proteins have dose-dependent negative effects on cell proliferation and cell survival. The observations that the C-terminal tail can function as a nuclear localization signal and that overexpression of wild type EF-G1 proteins can also reduce wing growth suggest a model, where EF-G1 functions as a retrograde signal to arrest the cell cycle, if the level of ATP synthesis in mitochondria is insufficient for proliferation. In this model, almost all wild type EF-G1 is imported into mitochondria, if they generate normal levels of ATP. However, if mitochondrial ATP synthesis is low or EF-G1 is overexpressed and import of EF-G1 proteins into mitochondria is a limiting step, some EF-G1 proteins can accumulate outside of mitochondria and translocate into the nucleus, where they inhibit cellular growth and proliferation. If mutant EF-G1 proteins are expressed in wild type cells, they likely bind to ribosomes and interfere with mitochondrial translation, which results in reduced ATP synthesis, which causes a decrease of EF-G1 import into mitochondria and accumulation of the protein outside of mitochondria in the nucleus (Figure 6I and O). In contrast, overexpression of wild type EF-G1 proteins is not expected to interfere with mitochondrial translation. They can only accumulate in the nucleus, if overexpression increases the level of wild type EF-G1 high enough to overwhelm mitochondrial import. Consistent with such a model, expression of wild type EF-G1 proteins is compared to missense mutant proteins not lethal and does not cause pattern defects (compare Figure 5 C and D). In addition, wild type transgenes without the C-terminal tail can rescue *ico* mutants as efficient as full-length transgenes (Table 1). However, wild type or mutant forms without the C-terminal tail have less severe effects on growth and patterning than the full-length proteins when expressed at high levels in wings (compare Figure 5C and E, 5D and 5F). Similarly, ubiquitous expression of two mutant transgenes with the C-tail is lethal, while expression of three transgenes without the tail is not. Taken together, these results show that the nuclear localization signal strongly contributes to the toxicity of the mutant EF-G1 proteins, suggesting that accumulation of EF-G1 in the nucleus is responsible for the observed defects.

Currently, the mechanism whereby nuclear EF-G1 proteins affect these tissues remains unclear. We have found that animals heterozygous for *ico* missense mutations suppress the overproliferation and pattern defects caused by expression of a constitutively activated form of the DPP type I receptor TKV in wings. Similarly, *ico* missense mutations reduce DPP signaling in embryos (Figure 1). In contrast, the deletion mutations that lack the C-terminal tail do not interact with DPP signaling in embryos or wings. The finding that only full-length EF-G1 proteins with the nuclear localization signal can inhibit growth and DPP signaling suggests that EF-G1 proteins have a function in the nucleus downstream of DPP signaling. However, it is not clear whether these effects are DPP specific or shared with other growth promoting pathways. Additional research using *Drosophila* as a model system should be able to provide better insights into the growth inhibitory mechanism of EF-G1.

### The role of EF-G1 and EF-G2 in flies and humans

Although *ico* is expressed in most tissues (Flybase and unpublished data), the lack of any visible defects in eye clones of *ico* deletion mutants, which lack the necessary domains for proper interactions with ribosomes and likely lack elongation activity, suggests that EF-G1 may not be required in all tissues. Since translation of mitochondria-encoded proteins is an essential process, this result also suggests that another protein can perform the function of EF-G1 in its absence. The best candidate for such a function is the EF-G2, which is present in all eukaryotic cells from yeast to humans. However, overexpression of EF-G2 cannot rescue *ico* mutants (Table 1). Similarly, human EF-G2 cannot compensate for the loss of EF-G1 in affected tissues of COXPD1 patients [5]. On the other hand, ubiquitous expression of EF-G1 cannot rescue the pupal lethal phenotype of flies with reduced EF-G2 levels due to gene silencing (unpublished results). A recent publication analyzing the functions of EF-G1 and 2 in bacteria and in liver tissues suggested that EF-G2 does not function in elongation but ribosomal recycling [16]. Taken together, these data indicate that EF-G1 and EF-G2 perform distinct functions.

Although EF-G1 and EF-G2 undeniably exhibit non-overlapping roles, the current data do not rule out the possibility that EF-G2 is an elongation factor. It is possible that EF-G2 can catalyze elongation but only in mitochondria of certain tissues. The finding that the loss of EF-G1 does not affect eye development can be explained if *Drosophila* EF-G2 assumes the function of elongation in the eye. If the elongation activity of EF-G2 is restricted to certain tissues, overexpression of EF-G2 is not expected to rescue *ico* mutants or substitute for EF-G1 in tissues affected in COXPD1 patients. This scenario would also explain why EF-G2 was shown to have no elongation activity in the liver [16], a tissue highly affected by the loss of EF-G1 [5].

In human patients, the loss of EF-G1 does not cause pathology in all tissues. In COXPD1 patients, the mutant EF-G1 protein was found to be present at reduced levels in mitochondria of non-affected tissues like the heart but non-detectable in mitochondria of tissues with severely reduced mitochondrial function like the liver [5]. Our rescue experiments show that the human mutant hEF-G1-N174S and hEF-G1-S321P proteins are stable and retain activity in flies. Taken together, these data suggest that EF-G1 import is reduced in all tissues of COXPD1 patients and that the levels of EF-G1 import indicates whether mitochondrial translation occurs sufficiently in a tissue.

This raises the question of why the import of mutant EF-G1 is reduced. If EF-G2 functioned as an elongation factors in tissues like the heart, mitochondrial translation and ATP synthesis would occur at normal levels, and EF-G1 would be imported into mitochondria and not accumulate in the nucleus. On the other hand, in tissues like the liver, where EF-G2 cannot function as an elongation factor, mitochondrial translation would decrease, ATP levels would drop, EF-G1 import into mitochondria would decrease and accumulation in the nucleus increase, which would further exacerbate the problem. Although this model is consistent with the current data, it is highly speculative at this moment, and additional experiments are required to further explore this hypothesis in flies and humans.

## Materials and Methods

### Mutant analysis

If not indicated otherwise, fly strains were obtained from the Bloomington stock center. The missense mutant allele *ico* ***^II032^*** was created by EMS mutagenesis [11]. The alleles *ico* ***^BA18^***, *ico* ***^GA1^***, and *ico* ***^GA25^*** were induced by EMS in a *black* and *cinnabar* background [12]. To analyze the cuticles of mutant animals, embryos were dechorionated, mounted in Hoyer's medium, and incubated at 65°C for 5 hours. The deletion alleles *ico* ***^Del EY1^*** and *ico* ***^Del EY2^*** were generated by mobilization of the P-element *EY05983*, isolated by complementation testing using the Exelixis lines *Df(2L)Exel8019*, *Df(2L)Exel6017*, *and Df(2L)Exel7031*, and confirmed through PCR using the primers: 5′TTTCCCTATTGCTCTCGCACG and 5′ACAGAGTTCTATCCCAGATGAG.

### Molecular biology

All alleles were sequenced by Davis Sequencing (Davis, CA). The transgenic constructs were sequenced by Retrogen, Inc. The sequence alignment was performed using the ClustalW function of the MacVector bioinformatics program (Figure 2). The accession number for the *CG4567* (*ico*) mRNA is NM135261 and NM 024996 for the human *EF-G1* (*GFM1)* mRNA. The annotated sequence of *CG31159 (Drosophila EF-G2)* is slightly different from our isolated cDNA, which has been submitted to NCBI.

The transgenic construct for wild type *ico* was created from a cDNA with a PCR extension from a *Drosophila* cDNA library [18] using the primers 5′ACAGAGTTCTATCCCAGATGAG and 5′GCGCCTCGAGCTAGTTCTTCTTTTTCTTCTTGTC, and cloned into the BglII and XhoI sites of *pUAST* [*14*].

The C-terminal deletion form CG4567ΔCstop was generated by PCR using the C-terminal primer GGCCTAGGCCAATCCTTGCGACTCC.

The human *EF-G1* constructs were generated using a clone from ATCC (#10436473). The human *EF-G1* cDNA was PCR amplified from this clone using the primers 5′GTGCGGTACCGGCAGCTGAACCCAC and 5′GCCTCTAGAGTCAGTCAACTCACAGTAAG, and the fragment was cloned into the Asp718 and XbaI sites of *pUAST2* [*15*].

A *Drosophila EF-G2* cDNA *(CG31159)* was amplified from an embryonic library [18] using the primers 5′TTTAGATCTGAAAATGCTGAAATATGCATGGC and 5′GGCCGCTAGCTATTCAAGGCCCTGTGCTCTG and cloned into the BglII and XbaI sites of *pUAST*.

The mutant *CG4567-G538E*, *hEF-G1-N174S*, and *hEF-G1-S321P* transgenic constructs were created by site-directed mutagenesis on the wild type constructs using the QuikChange method (Stratagene, La Jolla, CA).

The GFP and GST-tagged constructs were generated by cloning PCR-fragments of *GFP* and *GST* into *pUAST2* (NheI, TP, MssI, GFP or GST, NaeI, protein of interest, XbaI, BglII). Fusion proteins were generated by fusion of C-terminal fragment to a unique NaeI site introduced in *GFP* and *GST*. The mitochondrial target sequence was added by cloning a fragment in front of GFP or GST using NheI (in *pUAST2*) and MssI (in *GFP* and *GST*).

Transgenic constructs were sequenced by Retrogen and injected by Genetic Services. The transgenic constructs were tissue-specifically expressed using the GAL4 driver lines described in the text.

### Clonal analysis of ico alleles in the eye

In order to generate eye clones homozygous for *ico* mutations, crosses were performed as follows for each of the six *ico* mutant alleles: *white*, *eyeless*-*Flipase/Y*; *mini-white*, *cell-lethal(2L) FRT(2L)/CyO* males were crossed to *white/white*; *ico*, *FRT(2L)/CyO* females. Since the Flipase is located on the X-chromosome, recombination only occurs in the eyes of female progeny that was examined for the presence of white, non-colored cells derived from recombinant clones of homozygous *ico* mutants.

### Analysis of the subcellular localization of EF-G1 and 2 proteins in tissue culture cells

A *GAL4* cDNA was cloned into *pAcpA*, which contains the Actin5C promoter and polyA regions [19]. 2×10^6^ Schneider 2 (S2) cells were co-transfected with 1 µg of *actin5C*-*GAL4* and 1 µg of the *pUAST-EF-G1* and *pUAST-EF-G2* DNAs. After four days, the expression of GFP-tagged proteins was analyzed with a Nikon confocal microscope. Cells that express GST-tagged proteins were incubated after four days with DAPI (Sigma) and MitoTracker (Invitrogen M7512) for one hour, fixed with PBS/4% formaldehyde for 20 minutes, and detected using a FITC coupled anti-GST Antibody (GenWay 18-272-197537) with the confocal microscope.
